# Leiomyoma of the Cheek

**DOI:** 10.1155/2020/8474905

**Published:** 2020-02-19

**Authors:** Ricardo Roberto de Souza Fonseca, Mário Augusto Ramos Junior, Douglas Baruchi, Tabata Resque Beckmann Carvalho, Andresa Borges Soares, Silvio Augusto Fernandes de Menezes, João de Jesus Viana Pinheiro, Jorge Nazareno Ferreira Fadul

**Affiliations:** ^1^Laboratory of Virology, Institute of Biological Sciences, Federal University of Pará, Belém, PA, Brazil; ^2^Department of Oral Pathology, Barros Barreto Hospital, Belém, PA, Brazil; ^3^Department of Oral Pathology, São Leopoldo Mandic Institute and Research Center, Campinas, SP, Brazil; ^4^Department of Periodontology, University Center of State of Parã, Belém, PA, Brazil; ^5^Department of Periodontology, Pará State University, Belém, PA, Brazil; ^6^Department of Oral Pathology, Federal University of Pará, Belém, PA, Brazil

## Abstract

Leiomyomas are rare benign tumors that grow in the tunica media of smooth muscle cells. Leiomyomas occur most frequently in the uterus or gastrointestinal tract and only very rarely in the area of the cheek. This study reports on a rare case of a leiomyoma in the cheek of a 43-year-old woman, who presented with a well-circumscribed, asymptomatic, mobile swelling in the right cheek. This swelling was slightly purplish in color and measured approximately 4 cm × 3 cm. Surgical excision was the treatment of choice, and the diagnosis was based on histopathological and immunohistochemical stains, which were positive for actin and desmin and negative for AE1/AE3, CD34, and S100. The patient's follow-up, two years later, showed no recurrence, and she has been asymptomatic since the surgery.

## 1. Introduction

Leiomyomas are benign mesenchymal tumors arising from nonepithelial tissues, such as smooth muscle [1, 2]. These tumors are well-circumscribed neoplasms that are encountered frequently in the dermis, the gastrointestinal tract, and the female genital tract, in particular, the uterus [3, 4]. As smooth muscle cells are relatively rare in the oral cavity, in comparison with the gastrointestinal tract, oral leiomyomas are extremely rare, with an incidence of less than 1% of all benign, soft tissue tumors [5]. Leiomyomas are neoplasms which develop due to mutation that results in the loss of the growth regulation mechanisms of the smooth muscle cells [5, 6]. The development of this benign tumor depends on a complex interaction between hormones (estrogen, progesterone), growth factors, and cytokines, as well as genetic predisposition [7, 8]. The risk factors include middle age (fourth to sixth decades of life), dark skin, women with a history of chronic disease, including recurrent gynecological infections, and a high body mass index [9]. Leiomyomas are manifested clinically as slow-growing, asymptomatic lesions that are well-circumscribed and often purplish in color [10, 11]. Histologically, three types of leiomyoma can be distinguished: (a) leiomyoma (solid leiomyoma), (b) angioleiomyoma (vascular leiomyoma), and (c) epithelioid leiomyoma (leioblastoma) [12, 13]. The clinical aspects of leiomyomas are indistinguishable from other tumors located in the tongue or in the cheek, including pleomorphic adenomas, lymphangioma, pyogenic granulomas, and schwannomas [14, 15]. Diagnosis is based on histopathological examination, and surgical excision is the treatment of choice, with recurrence being extremely rare [16, 17]. The present study describes a rare case of a leiomyoma in the cheek of a 43-year-old woman.

## 2. Case Report

**Figure 1:**
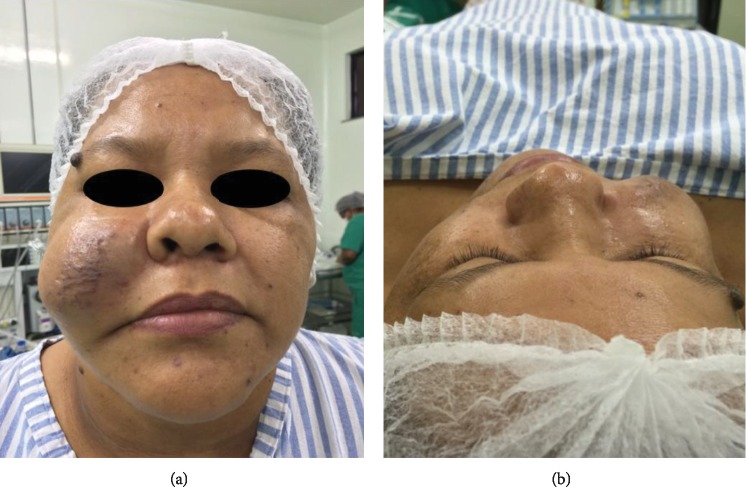
(a, b) Clinical aspect of the lesion, showing the large size of the growth.

A 43-year-old woman, a nonsmoker, with no medical antecedents was referred to the Departments of Oral and Maxillofacial Surgery of a dental college in northern Brazil for evaluation of a well-circumscribed, solitary, asymptomatic, mobile mass in the right cheek, which was slightly purplish in color, causing facial asymmetry (Figures 1(a) and 1(b)).

**Figure 2:**
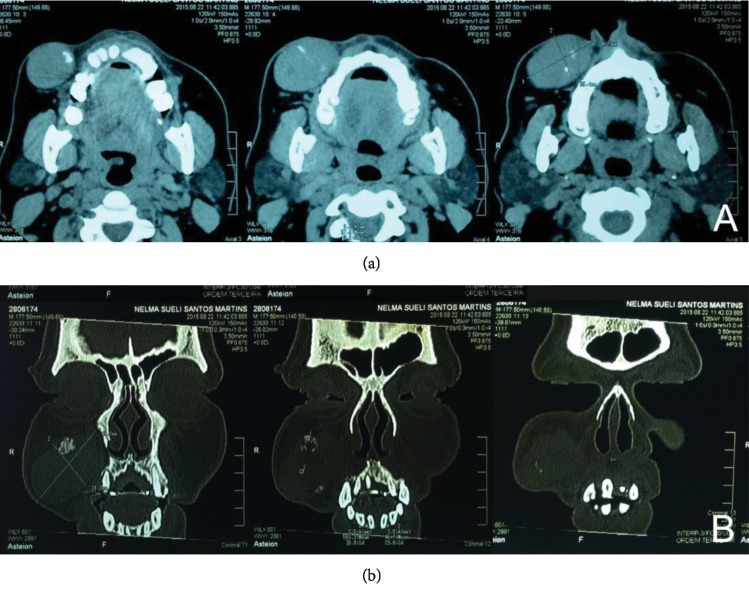
(a) Axial plane and (b) coronal plane, showing the lesion as a dense mass of soft tissue with defined contours and foci of calcification.

The patient reported having had a swelling in the right cheek over the previous two years. She also reported that she was not on any drug therapy and did not consume alcohol regularly. Physical and oral examination revealed a well-demarcated, palpable, hard elastic mass measuring approximately 4 cm × 3 cm, located in the submucosal layer of the right cheek. The overlying mucosa appeared clinically normal and was not ulcerated. A CT scan revealed a homogenous mass with well-defined margins and no evidence of maxillary sinus infiltration or bone resorption (Figures 2(a) and 2(b)).

**Figure 3:**
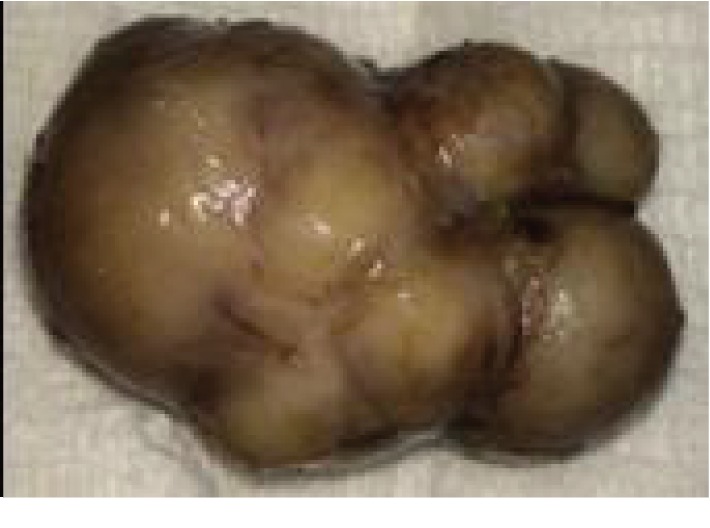
Macroscopic aspect of the leiomyoma.

**Figure 4:**
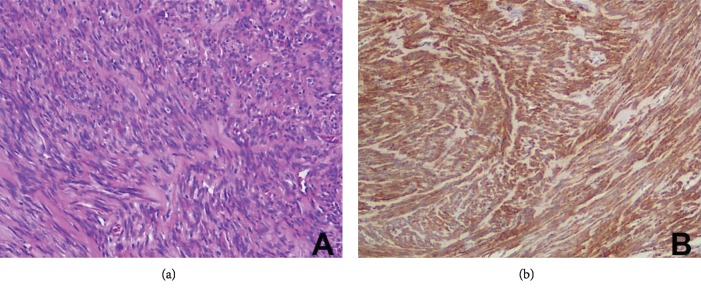
(a) Proliferation of spindle cells arranged in interlocking bundles in a monotonous pattern of fascicles (hematoxylin and eosin stain; original magnification 20x). (b) Immunohistochemical stain showing strong positivity for desmin (original magnification 20x).

**Figure 5:**
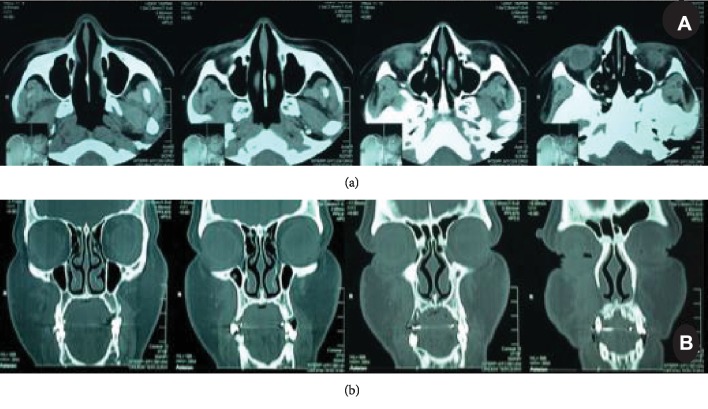
(a) Axial plane and (b) coronal plane, showing the postoperative aspects of the affected region.

**Figure 6:**
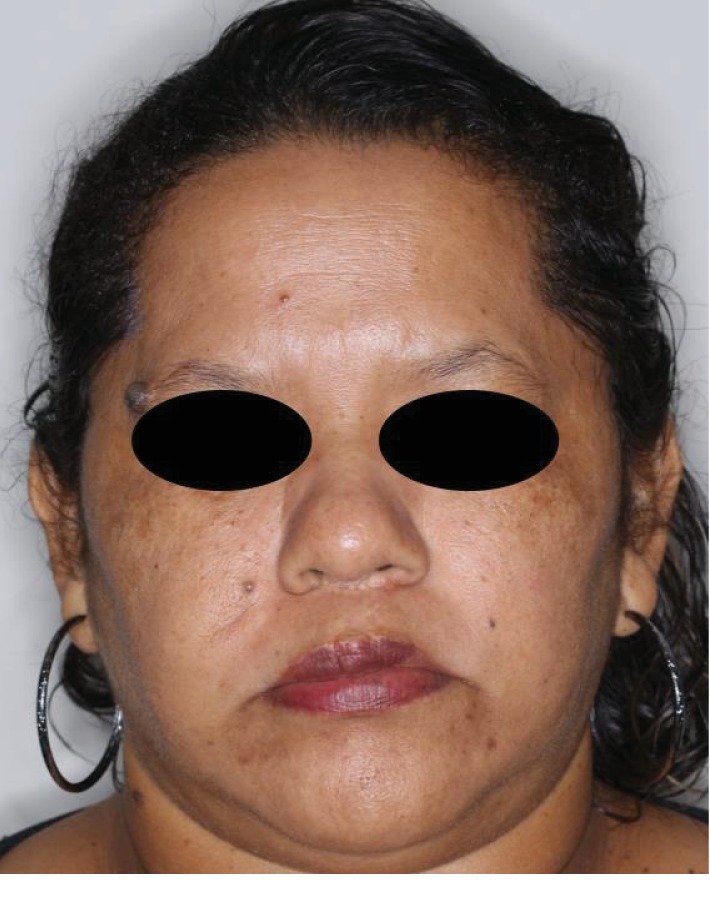
Two-year follow-up with no signs of recurrence.

The patient underwent an excisional biopsy (Figure 3). Microscopic examination revealed uniform spindle-shaped cells with elongated nuclei, eosinophilic cytoplasm; several blood vessels lined with a thin layer of endothelial cells were embedded within the lesion (Figure 4(a)). Immunohistochemical stains with monoclonal antibodies against actins, desmin, vimentin, cytokeratins AE1/AE3, CD34, and the S100 protein (Figure 4(b)) were performed. Stains for vimentin, desmin, muscle-specific actin, and smooth muscle actin were positive while stains for cytokeratins AE1/AE3, EMA, S100, and CD34 were all negative in the tumor cells. The histopathological diagnosis was leiomyoma of the cheek. The follow-up examinations over 2 years after the procedure showed no evidence of recurrence (Figures 5(a), 5(b), and 6).

## 3. Discussion

Baden et al. described leiomyomas as a benign neoplasm that arises in the smooth muscle [18, 19]. As reported in several previous reports [18–28], leiomyomas can be found in areas with an abundance of smooth muscle, such as the uterine or gastrointestinal tracts and the dermis. Given the lack of smooth muscle in the oral cavity, in particular in the cheek, palate, or tongue, the only available substrates in this area would be blood vessels, circumvallate papillae, and heterotopic smooth muscle [20]. The pathogenesis of leiomyoma in the oral cavity is unclear, although oral trauma, venous stasis, hormonal changes, and genetic alterations can be implicated [20, 21].

Previous studies [22–24] have reported leiomyomas of the oral cavity primarily in the lips, followed by the palate, the buccal space, the mandible, the tongue, and the gingiva, while the cheek is the least frequent site of leiomyomas in the oral cavity [25, 26]. Given this, the case of leiomyoma reported here in the cheek of a 43-year-old female can be considered to be relatively rare. The exceptional nature of the present case is reinforced by the fact that most cases of leiomyoma in the oral cavity are relatively small, that is, typically less than 2 cm in diameter, nodular, and slow-developing. The cheek leiomyoma described in the present study was larger than any benign smooth muscle tumor reported previously [27, 28]. Clinically, it is difficult to distinguish leiomyomas of the cheek from other lesions or tumors of the oral cavity, such as pleomorphic adenoma, lymphangioma, fibroma, lipoma, or pyogenic granuloma [20–24]. However, angioleiomyomas, unlike leiomyomas, appear reddish blue in color and are soft and compressible on palpation. The benign characteristics of leiomyomas include the absence of mitoses and necrosis, as well as cellular atypia and pleomorphism in histopathological evaluation [25–28]. The diagnosis of leiomyoma is based on positivity for desmin, smooth muscle actin, and muscle-specific actin and negativity for the S100 protein [28].

Leiomyomas recur only very rarely, although prognosis depends on the completeness of the surgical excision of the tumor. In one case, an angioleiomyoma was excised from the region of the hard palate, but 9 weeks after the first procedure, the authors noted the recurrence [27]. In the second case of an angioleiomyoma in the region of the hard palate, recurrence was noted two weeks after the first excision [28].

## 4. Conclusion

A leiomyoma of the cheek is a rare benign neoplasm. We report a case of a leiomyoma located in the buccal mucosa of the oral cavity. Tumors of this type are asymptomatic and may develop for months or even years. The diagnosis of this lesion requires histopathological evaluation.
